# Brain Injury and Stem Cell Replacement

**DOI:** 10.1155/2018/5173494

**Published:** 2018-04-05

**Authors:** Hailiang Tang, Yao Li, John Zhang

**Affiliations:** ^1^Department of Neurosurgery, Huashan Hospital, Fudan University, Shanghai 200040, China; ^2^Med-X Research Institute, Shanghai Jiaotong University, Shanghai 200030, China; ^3^Center for Neuroscience Research, Loma Linda University School of Medicine, 11175 Campus St., Loma Linda, CA 92350, USA

Currently, stem cell is still the research hotspot in neuroscience due to its important role for regeneration medicine. Stem cells could aid neurogenesis and functional recovery in animals and human beings after brain injury, which was always caused by stroke or traumatic brain injury (TBI). Neural stem cell (NSC) replacement is considered a very promising therapy strategy for brain injury. Many studies had been conducted in animal experiments and clinical trials.

The purpose of this special issue is to provide readers with an overall outlook of the recent advances in stem cell replacement for brain injury. The topics cover neural stem cells and other stem cells, traumatic brain injury and ischemic stroke, overview of stem cell therapy for brain injury, mechanism of stem cell therapy, and stem cell tracking in the host brain.

This special issue published 17 excellent papers regarding the above specific topics, and the details are summarized below.

Stem cell replacement for brain injury is a very promising therapy, but how to evaluate the therapeutic effect is still difficult. Y. Zheng et al. provided the way on how to track these stem cells after treatment; they overall reviewed the advances on stem cell tracking for brain injury and summarized the current techniques applied for stem cell tracking. With regard to ischemic stroke, W. Xu et al. thoroughly reviewed the role of stem cells for this kind of stoke, and Y. Zhang and H. Yao discussed transplanted stem cells for it. Moreover, Y. Lu et al. discussed the adult neurogenesis after stroke therapy. In another group, C. Reis et al. explored the stem cell therapy options for ischemic stroke, which were very instructive. This team also reviewed the mechanism of stem cell therapy for traumatic brain injury. The two kinds of brain injury (TBI and stroke) were both covered. And what is more, M. Cui et al. interestingly described the electromagnetic regulation of neural stem cells for brain injury.

The guest editors hope this special issue provides readers with helpful information of recent advances in stem cell therapy for brain injury and may stimulate interest for further research in this area.

